# Editorial: Country profile of the epidemiology and clinical management of early childhood caries, volume II

**DOI:** 10.3389/fpubh.2023.1201899

**Published:** 2023-06-06

**Authors:** Morenike Oluwatoyin Folayan, Francisco Ramos-Gomez, Wael Sabbah, Maha El Tantawi

**Affiliations:** ^1^Early Childhood Caries Advocacy Group, Winnipeg, MB, Canada; ^2^Africa Oral Health Network, Alexandria, Egypt; ^3^Oral Health Initiative, Nigerian Institute of Medical Research, Lagos, Nigeria; ^4^Department of Child Dental Health, Obafemi Awolowo University, Ile-Ife, Nigeria; ^5^SOD-Division of Preventive and Restorative Oral Health Sciences, University of California, Los Angeles, Los Angeles, CA, United States; ^6^Faculty of Dentistry, Oral and Craniofacial Sciences, King's College London, London, United Kingdom; ^7^Department of Pediatric Dentistry and Dental Public Health, Faculty of Dentistry, Alexandria University, Alexandria, Egypt

**Keywords:** inequality, elimination, dental caries, inequity, collaboration, human right

Early childhood caries (ECC) is a non-communicable disease of global public health importance. It affects more than 530 million children all over the world. ECC prevalence is high in some countries and low in others with little explanations for the observed disparities. For example, in this Research Topic, Chouchene et al. highlighted that the prevalence of ECC was 20% for 3–5-year-olds in Tunisia; Guan et al. highlighted that the prevalence was to 63.1% for 4–5-year-old in Guizhou Province, China, while Liu et al. highlighted that the prevalence was 74.3% in 3–5-year-olds in Xiangyun, China; and Şengül et al. showed the prevalence was 73.3% for 4–5-year-olds in Turkey.

There seems to be consensus, however, that ECC is a disease of inequality with a heavier burden in lower-income countries and among children from lower socioeconomic backgrounds. Yet, the global profile of ECC is not so distinct and cannot be stratified completely by country income profile. For example, sub-Saharan Africa is one of the poorest regions in the world (1). However, in this region, Gambia, which a low-income country, has one of the lowest levels of ECC prevalence in the world while the Central African Republic, the Democratic Republic of Congo and Gabon which have the same income level as Gambia, have one of the highest levels of prevalence of ECC in the world (2). Similarly, Nigeria, a low-middle-income country, has the second lowest prevalence of ECC in children younger than 36-months-old and the second lowest prevalence of ECC in children 36–71 months old after Denmark (3).

Our understanding of the factors driving inequity in ECC distribution may have been limited by the theoretical lens by which we used to study ECC. Studies on the social and structural drivers of ECC are limited and so are studies that use the human right approach. Most studies on ECC explore individual and household factors. Further investigation is needed into studies that explore the impact of food and food policies on the risk of ECC as highlighted by Amalia et al.. A study by Albrecht showed a link between soil fertility and epidemiology of caries in the US. This study noted that *soils with a high capacity for protein production, because of their high mineral fertility, are the soils that have also grown better teeth* (4). Studies of the land (sustainable development goal 15), food and dietary diversity may improve understanding of ECC and how to proceed to eliminate this public health threat. As identified in this Research Topic, Wang et al. showed that dietary diversity and vegetable meals are associated with lower risk of ECC but grain diet is associated with higher ECC risk. The study of land and food may help improve our understanding of culture and its impact on oral health. Culture may be a better tool to understand the distribution of ECC than country income levels.

Studies of health systems and how they can support the control of ECC are also important. Integrating ECC management into primary health care and pediatric healthcare delivery systems in every country can improve children's access to oral healthcare. In this Research Topic, Shmueli et al. highlighted that collaborating with a wide range of healthcare workers to deliver sustainable oral healthcare tailored to the needs of local communities will be required to promoting oral and dental health in early childhood in Israel. Prior to this issue highlighted in this Research Topic, other authors had highlighted the need to establish a collaborative partnership between oral health care providers and community-based oral health workers is needed to to reach hard-to-reach populations (5); and supporting interprofessional education and collaborative practice between oral health, medical and other pediatric primary care providers is needed (6).

The study of ECC may also need new methodologies. Of interest is the use of single question self-measure as an indicator of ECC. Experts are skeptical about the validity of single-item measures to measure cognitive and affective outcomes. Yet, single-item measures can provide valid and reliable assessment of important phenomena just like their multi-item counterparts (7). Single item measures allow the conduct of shorter surveys, reduce research costs and improve the quality of research participants' engagement leading to greater survey effectiveness. Single item measures may also be more suited to certain populations (8). In this Research Topic, Imes et al. demonstrated how maternal assessment of oral health using a single-item measure was indicative of caries and untreated caries.

In effect, governments, global actors and research stakeholders need to do more to reduce the ECC burden. We can collectively do more if we continue to show evidence on “why” the prevalence of ECC continue to be high, and “how” to reduce this prevalence and mitigate its impact. Hopefully, the generation of new evidence to drive a collective global response for the ECC can help us reach a point where the elimination of untreated ECC becomes a possibility. The elimination of untreated ECC is a worthy target considering its significant impact on children' growth, development, quality of life, and wellbeing.

## Author contributions

MF conceptualized the Research Topic, wrote the first draft of the manuscript, and developed the final version of the manuscript. FR-G, WS, and ME edited the manuscript and agreed to the final version of the manuscript. All authors contributed to the article and approved the submitted version.

## References

[1] Schoch M, Laker, C,. The Number of Poor People Continues to Rise in Sub-Saharan Africa, Despite a Slow Decline in the Poverty Rate. World Bank Blog. (2020). Available online at: https://blogs.worldbank.org/opendata/number-poor-people-continues-rise-sub-saharan-africa-despite-slow-decline-poverty-rate (accessed April 7, 2023).

[2] WHO Ending Childhood Dental Caries: WHO Implementation Manual. Geneva: WHO (2019).

[3] El Tantawi M Folayan MO Mehaina M Vukovic A Castillo JL Gaffar BO. Prevalence and data availability of early childhood caries in 193 united nations countries, 2007-2017. Am J Public Health. (2018) 108:1066–72. 10.2105/AJPH.2018.304466 29927650 PMC6050821

[4] Albrecht WMA. Our Teeth and Our Soils. Columbia, MO: University of Missouri College of Agriculture Agricultural Experiment Station Circular. (1948). p. 333.

[5] Agbor AM Azodo CC Naidoo S. The Oral Health workforce in Cameroon; the past, the present and the future. Afri J Oral Health. (2018) 7:11–5. 10.4314/ajoh.v7i2.172404

[6] Ramos-Gomez F Askaryar H Garell C Ogren J. Pioneering and interprofessional pediatric dentistry programs aimed at reducing oral health disparities. Front Public Health. (2017) 5:207. 10.3389/fpubh.2017.00207 28856133 PMC5557784

[7] Allen MS Iliescu D Greiff S. Single item measures in psychological science. Eur J Psycholog Assess. (2022) 38:a000699. 10.1027/1015-5759/a000699

[8] Hoeppner BB Kelly JF Urbanoski KA Slaymaker V. Comparative utility of a single-item versus multiple-item measure of self-efficacy in predicting relapse among young adults. J Subst Abuse Treat. (2011) 41:305–12. 10.1016/j.jsat.2011.04.005 21700411 PMC3315352

