# Corrigendum: Editorial: Country profile of the epidemiology and clinical management of early childhood caries, volume II

**DOI:** 10.3389/froh.2023.1242565

**Published:** 2023-12-11

**Authors:** Morenike Oluwatoyin Folayan, Francisco Ramos-Gomez, Wael Sabbah, Maha El Tantawi

**Affiliations:** ^1^ Early Childhood Caries Advocacy Group, Winnipeg, MB, Canada; ^2^ Africa Oral Health Network, Alexandria, Egypt; ^3^ Oral Health Initiative, Nigerian Institute of Medical Research, Lagos, Nigeria; ^4^ Department of Child Dental Health, Obafemi Awolowo University, Ile-Ife, Nigeria; ^5^ SOD-Division of Preventive and Restorative Oral Health Sciences, University of California, Los Angeles, CA, United States; ^6^ Faculty of Dentistry, Oral and Craniofacial Sciences, King’s College London, London, United Kingdom; ^7^ Department of Pediatric Dentistry and Dental Public Health, Faculty of Dentistry, Alexandria University, Alexandria, Egypt

**Keywords:** inequality, elimination, dental caries, inequity, collaboration, human right

A Corrigendum on Editorial: Country profile of the epidemiology and clinical management of early childhood caries, volume II By Folayan MO, Ramos-Gomez F, Sabbah W, El Tantawi M (2023). Front. Public Health. 11:1201899. doi: 10.3389/fpubh.2023.1201899

**Text Corrections**

A correction has been made to paragraph 2. This sentence previously stated:

“have one of the highest levels of prevalence of ECC in the world”

The corrected sentence appears below:

“have one of the highest levels of ECC prevalence in the world”

A correction has been made to paragraph 3. This sentence previously stated:

“inequity in ECC distribution”

The corrected sentence appears below:

“inequity in ECC prevalence”

A correction has been made to paragraph 3. This sentence previously stated:

“Further investigation is needed into studies that explore the impact of food and food policies on the risk of ECC”

The corrected sentence appears below:

“Further investigation is needed to explore the impact of food and food policies on the risk of ECC”

A correction has been made to paragraph 3. This sentence previously stated:

“Culture may be a better tool to understand the distribution of ECC than country income levels”

The corrected sentence appears below:

“Culture may be a better tool to understand the inequity in ECC prevalence than country income levels”

A correction has been made to paragraph 4. This sentence previously stated:

“Prior to this issue highlighted in this Research Topic, other authors had highlighted the need to establish a collaborative partnership between oral health care providers and community-based oral health workers is needed to to reach hard-to-reach populations (5); and supporting interprofessional education and collaborative practice between oral health, medical and other pediatric primary care providers is needed (6)”

The corrected sentence appears below:

“Prior to now, other authors had highlighted the need to establish collaborative partnerships between oral health care providers and community-based oral health workers to be able to reach hard-to-reach populations (5); and supporting interprofessional education and collaborative practice between oral health, medical and other pediatric primary care providers (6)”

A correction has been made to paragraph 5. This sentence previously stated:

“In this Research Topic, Imes et al. demonstrated how maternal assessment of oral health using a single-item measure was indicative of caries and untreated caries.”

The corrected sentence appears below:

“In this Research Topic, Imes et al. demonstrated how maternal assessment of oral health was indicative of caries and untreated caries using a single-item measure.”

The authors apologize for these errors and state that this does not change the scientific conclusions of the article in any way. The original article has been updated.

